# Transcriptomic Profile of Lymphovascular Invasion, a Known Risk Factor of Pancreatic Ductal Adenocarcinoma Metastasis

**DOI:** 10.3390/cancers12082033

**Published:** 2020-07-24

**Authors:** Hideo Takahashi, Eriko Katsuta, Li Yan, Yoshihisa Tokumaru, Matthew H.G. Katz, Kazuaki Takabe

**Affiliations:** 1 Department of Surgical Oncology, Roswell Park Comprehensive Cancer Center, Buffalo, NY 14263, USA; hideo.takahashi@roswellpark.org (H.T.); eriko.katsuta@roswellpark.org (E.K.); Yoshihisa.Tokumaru@roswellpark.org (Y.T.); 2 Department of Biostatistics and Bioinformatics, Roswell Park Comprehensive Cancer Center, Buffalo, NY 14263, USA; li.yan@roswellpark.org; 3 Department of Surgical Oncology, Graduate School of Medicine, Gifu University, Gifu 501-1194, Japan; 4 Department of Surgical Oncology, The University of Texas MD Anderson Cancer Center, Houston, TX 77030, USA; mhgkatz@mdanderson.org; 5 Department of Surgery, University at Buffalo Jacobs School of Medicine and Biomedical Sciences, the State University of New York, Buffalo, NY 14260, USA; 6 Department of Breast Surgery and Oncology, Tokyo Medical University, Tokyo 160-8402, Japan; 7 Department of Surgery, Yokohama City University, Yokohama 236-0004, Japan; 8 Department of Surgery, Niigata University Graduate School of Medical and Dental Sciences, Niigata 951-8510, Japan

**Keywords:** lymphovascular invasion, pancreatic cancer, TCGA

## Abstract

Lymphovascular invasion (LVI) is an aggressive pathologic feature and considered a risk factor for distant metastasis. We hypothesized that pancreatic ductal adenocarcinomas (PDACs) with LVI are associated with shorter survival, as well as aggressive cancer biology and lymphangiogenesis in transcriptomic analysis. Utilizing the cancer genome atlas (TCGA)-PDAC cohort, we found that positive LVI was significantly associated with positive perineural invasion (PNI) (*p* = 0.023), and higher American Joint Committee on Cancer (AJCC) T (*p* = 0.017) and N (*p* < 0.001) categories. Furthermore, positive LVI was associated with shorter overall survival (OS) (*p* = 0.014) and was an independent risk factor of poor OS. Although there was no association between LVI status and lymphangiogenesis, epithelial-mesenchymal transition (EMT), or metastasis-related genes, Gene Set Enrichment Analysis revealed a strong association with cell-proliferation-related gene sets such as mitotic spindles (Normalized enrichment score (NES) = 1.76, *p* = 0.016) and G2/M checkpoints (NES = 1.75, *p* = 0.036), as well as with transforming growth factor beta (TGF-beta) signaling (NES = 1.61, *p* = 0.043), which is a known mechanism of metastasis in PDACs. In conclusion, positive LVI was an independent risk factor of poor OS in PDACs. We found that PDACs with LVI were possibly associated with accelerated cell proliferation and enhanced TGF-beta signaling independent of lymphangiogenesis. Transcriptomic profiling elucidates more precise tumor biology of LVI-positive PDACs.

## 1. Introduction

Pancreatic ductal adenocarcinoma (PDAC) will become the second leading cause of cancer-related deaths in the United States by 2030 [1,2]. The exact reason for the increased incidence of PDACs is unclear; however, some risk factors for PDACs are being highlighted, such as the increasing rate of obesity, the increasing prevalence of diabetes, and chronic pancreatitis related to alcohol intake [3]. The overall 5-year survival rate remains less than 10% despite multidisciplinary cancer treatment advances in the last decade, as the majority of the patients present with locally advanced or metastatic diseases that preclude them from potentially curative resection [3]. Even with curative operations, up to 80% of patients recur in 1–2 years postoperatively [4,5]. Most risk factors for recurrence in PDAC are pathologically proven, such as lymph node metastases, tumor size, histologic grade, resection margin status, and lymphovascular and perineural invasion (PNI) [5,6,7,8]. Lymphovascular invasion (LVI) is pathologically defined as neoplastic invasion into the vascular or lymphatic vessels [9], and PNI as invasion into the perineural space [10]. Positive LVI and PNI are pathologic aggressive features of cancer and considered to be risk factors for recurrence and distant metastases in various types of malignancies [11,12,13,14,15]. LVI and PNI are commonly positive in PDAC, and have been reported in as many as 50–75% and 75–100% of specimens, respectively [5,7,16,17].

There have been multiple studies reporting an association between clinical outcome and pathological features, such as LVI and PNI in patients with PDAC [5,7,14,15]. Our group recently found that increased vascularity (high expression of CD31) might be associated with better prognoses in patients with PDAC, possibly due to better delivery of tumor-specific immune cells [18], using the bioinformatics analysis of transcriptomic profiles [19,20,21,22,23,24,25,26,27]. Overexpression in vascular endothelial growth factors (VEGFs) and podoplanin (PDPN) have been speculated to be associated with LVI in PDAC [28,29,30], although specific transcriptomic profiles of PDACs with positive LVI are scarce in the literature. Previous reports in other cancers suggested that positive LVI is associated with extracellular matrix (ECM) degradation, in which matrix metalloproteinases (MMPs) play key roles [31,32,33]. Additionally, increased lymphatic vessel density was reported to correlate with positive LVI and lymph node metastasis in breast cancer [34]. We hypothesized that PDAC with positive LVI is associated with lymphangiogenesis and other aggressive cancer biology in transcriptomatic analysis, resulting in lymph node metastasis and shorter survival. Employing a publicly available large data set, the Cancer Genome Atlas (TCGA), we investigated the impact of LVI status on PDAC patient survival, as well as the transcriptomic characteristics of LVI-positive PDACs.

## 2. Results

### 2.1. LVI Status Was Associated with PNI, American Joint Committee on Cancer (AJCC) T and N Categories in PDAC

**Figure 1:**
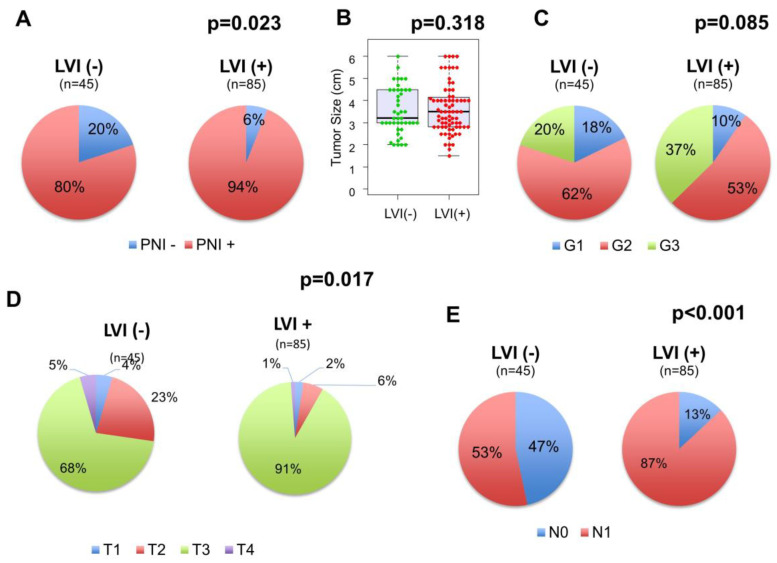
The association between lymphovascular invasion (LVI) status and clinicopathological features in pancreatic ductal adenocarcinomas (PDACs). (**A**) The distribution of perineural invasion (PNI) status in the LVI-negative and positive tumors, (**B**) Tumor size comparison between the LVI-negative and positive tumors, (**C**) The distribution of histological grade in the LVI-negative and positive tumors, (**D**) The distribution of AJCC T categories in the LVI-negative and positive tumors, (**E**) The distribution of American Joint Committee on Cancer (AJCC) N categories in the LVI-negative and positive tumors.

Out of 154 patients in the TCGA cohort, 130 (84.4%) and 123 (79.9%) had LVI and PNI information available, respectively. Among these patients, the number of PDACs with positive LVI and PNI were 85 (65.4%) and 109 (89.1%), respectively, similar to the previous reports [5,7,16]. The proportion of PNI-positive PDACs is higher in the LVI-positive tumors than in the LVI-negative tumors (*p* = 0.023, Figure 1A). Additionally, while tumor size (3.5 vs. 3.2 cm, *p* = 0.318, Figure 1B) or distribution of histological grade (*p* = 0.085, Figure 1C) were not different between the LVI-positive and negative tumors, a higher proportion of advanced AJCC T (*p* = 0.017, Figure 1D) and N (*p* < 0.001, Figure 1E) categories was observed in the LVI-positive tumors. There were no other statistically significant differences between LVI status and any of the features analyzed, including age, sex, tumor location, residual tumor status, and history of adjuvant radiation treatment (Table 1).

### 2.2. LVI Status Was Associated with Worse Prognosis in PDAC

**Figure 2:**
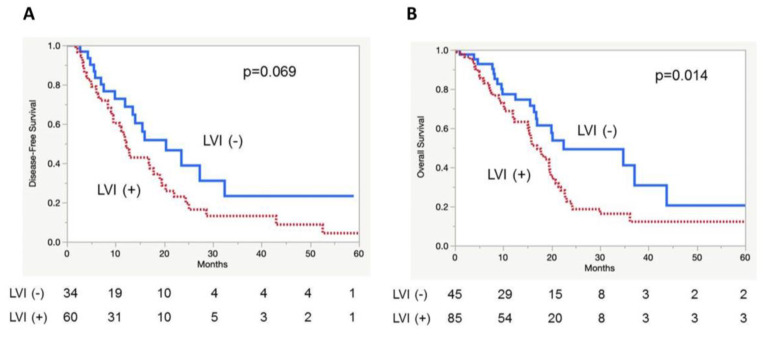
Kaplan-Meier curves depicting patient disease-free survival (DFS) and overall survival (OS) based on LVI status in PDACs. (**A**) DFS, (**B**) OS.

Survival analysis revealed that, although it did not reach statistical significance, patients with positive LVI tumors tended to have a shorter median disease-free survival (DFS) compared with patients without LVI (median DFS time: 12.2 vs. 20.4 months, *p* = 0.069, Figure 2A). Furthermore, patients with LVI-positive tumors had a significantly shorter median overall survival (OS) compared with patients with LVI-negative tumors (median OS time: 17.0 vs. 22.5 months, *p* = 0.014, Figure 2B). Univariate analysis showed that residual tumor status (*p* = 0.021), adjuvant radiation (*p* = 0.002), and positive LVI status (*p* = 0.011) were associated with OS. Multivariate analysis revealed that positive LVI was independently associated with shorter OS (*p* = 0.012, Table 2). Positive LVI was found to affect clinical outcome, which is in agreement with the previous reports [14,15]. LVI was significantly associated with shorter OS in the TCGA cohort, demonstrating that the TCGA-PDAC cohort is a reliable cohort that follows a similar trend as the previous reports in the literature.

### 2.3. Positive LVI Was Not Associated with Lymphangiogenesis, Angiogenesis, or Extracellular Matrix (ECM) Degradation Related Genes in PDAC

**Figure 3:**
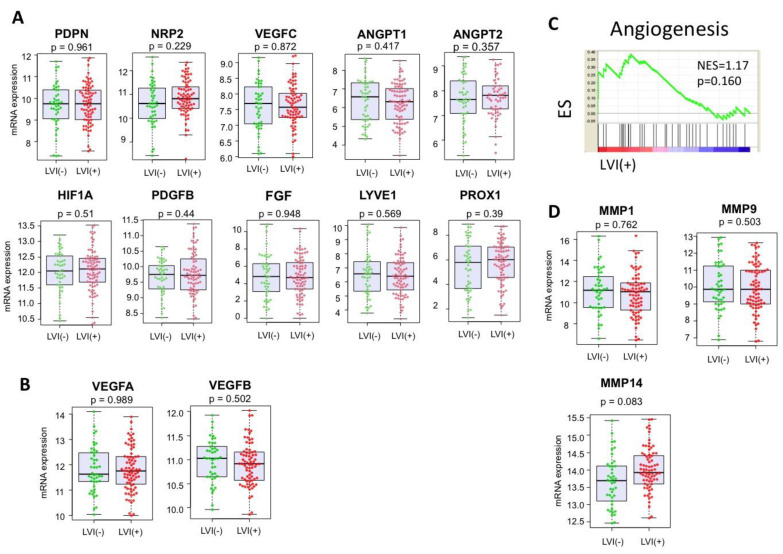
The associations of LVI status and lymphangiogenesis, as well as extracellular matrix (ECM) degradation in PDACs. (**A**) Gene expression comparison involved in lymphangiogenesis between the LVI-negative and positive tumors. (**B**) Gene expression comparison involved in angiogenesis between the LVI-positive and negative tumors. (**C**) Gene set enrichment analysis (GSEA) of the angiogenesis gene set, comparing the LVI-positive and negative tumors. (**D**) Gene expression comparison involved in ECM degradation between the LVI-positive and negative tumors. PDPN = podoplanin; NRP2 = neuropilin-2; VEGF = vascular endothelial growth factor; ANGTP = Angiopoietins; HIF = hypoxia-inducible factor; PDGF = platelet-derived growth factor; FGF = fibroblast growth factor; LYVE = lymphatic vessel endothelial hyaluronan receptor; PROX = prospero homeobox protein; ES = enrichment score; NES = normalized enrichment score; MMP = matrix metalloproteinase.

Given the previous reports that demonstrated higher expression of vascular and lymphatic angiogenesis-related genes in the LVI-positive tumors in breast and colon cancer [30,34,35], we speculated that vascular and lymphatic angiogenic genes might be upregulated in the LVI-positive PDACs. However, contrary to the previous reports, LVI was not associated with the expressions of lymphangiogenesis-related genes, such as PDPN, neuropilin-2 (NRP2), vascular endothelial growth factor (VEGF) C, Angiopoietins (ANGPT1 and ANGPT2), hypoxia-inducible factor (HIF)-1α, platelet-derived growth factor (PDGF)-BB, fibroblast growth factor (FGF), lymphatic vessel endothelial hyaluronan receptor (LYVE)-1, and prospero homeobox protein (PROX) 1 (Figure 3A), as well as vascular-angiogenesis-related genes, such as VEGFA and VEGFB (Figure 3B). Further, with gene set enrichment analysis (GSEA), the angiogenesis gene set was not enriched in the LVI-positive PDACs (normalized enrichment score (NES) = 1.17, *p* = 0.160, Figure 3C). Next, we compared the expression levels of genes involved in ECM degradation, such as matrix metalloproteinase (MMP)1, MMP9, and MMP14, which were previously reported to be elevated in LVI-positive breast cancers [31,32,33]. However, none of the MMP expressions were significantly different between the LVI-positive and negative PDACs (Figure 3D).

### 2.4. Positive LVI Was Associated with Promoted Cell Cycles and Enhanced Transforming Growth Factor (TGF)-Beta Signaling in PDAC

**Figure 4:**
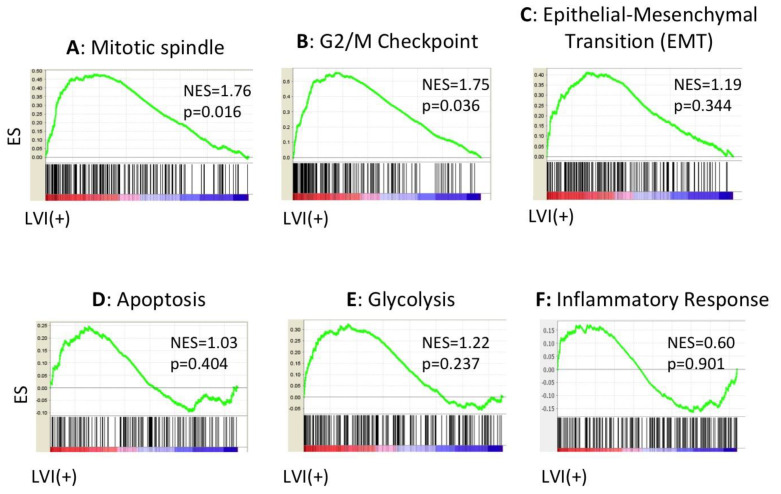
The associations of the LVI status and crucial functions in tumor growth in PDACs, analyzed by GSEA. (**A**) Enrichment plot of Mitotic spindle, (**B**) G2/M checkpoint, (**C**) epithelial-mesenchymal transition (EMT), (**D**) apoptosis gene sets, (**E**) glycolysis, and (**F**) inflammatory response.

**Figure 5:**
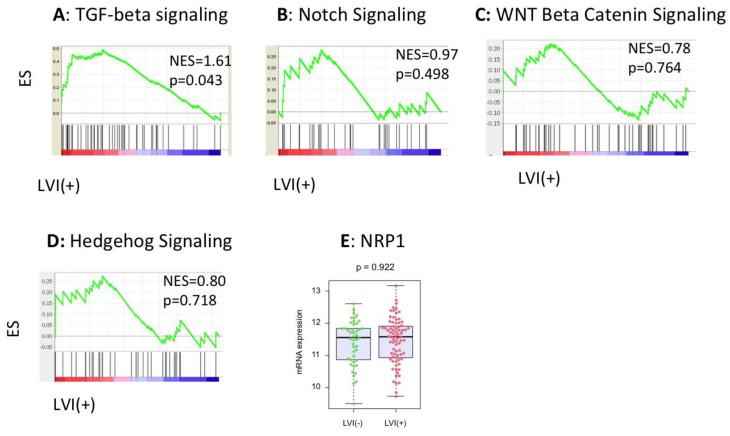
The association of LVI status and commonly altered signaling pathways in PDACs, analyzed by GSEA. (**A**) TGF-beta, (**B**) Notch, (**C**) WNT-beta catenin, and (**D**) Hedgehog pathway gene sets. (**E**) The association between LVI status and NRP1 expression. TGF = transforming growth factor; NRP1 = neuropilin-1.

Given that the positive LVI status was associated with increased lymph node metastases and a short median OS, we hypothesized that there are associations between LVI and aggressive cancer features, such as epithelial-mesenchymal transition (EMT), tumor inflammation, metabolism, cell proliferation, cancer stem cell maintenance, and several other important signaling pathways in PDACs. As Jones et al. reported that there were several core signaling pathways altered in most PDACs, including transforming growth factor beta (TGF-beta), Notch signaling, WNT-beta catenin, and Hedgehog pathways [36], we suspected that a positive LVI status might associate with these pathways. GSEA revealed that cell-cycle-related gene sets, including mitotic spindle (NES = 1.76, *p* = 0.016, Figure 4A) and G2/M checkpoint (NES = 1.75, *p* = 0.036, Figure 4B), were significantly enriched in the LVI-positive PDACs, whereas the LVI-positive PDACs did not enrich EMT (NES = 1.19, *p* = 0.344, Figure 4C), apoptosis (NES = 1.03, *p* = 0.404, Figure 4D), glycolysis (NES = 1.22, *p* = 0.237, Figure 4E), or the inflammatory response gene set (NES = 0.60, *p* = 0.901, Figure 4F). Additionally, there were no associations with cancer stem cell regulation or maintenance genes, including Forkhead box protein Q1 (FOXQ1), B cell-specific Moloney murine leukemia virus integration site 1 (BMI1), and Enhancer of zeste homolog 2 (EZH2) [37] (Supplemental Figure S1). Among the reported altered core signaling pathways, the TGF-beta signaling gene set was enriched in the tumors with positive LVI (NES = 1.61, *p* = 0.043, Figure 5A). However, Notch signaling (NES = 0.97, *p* = 0.498, Figure 5B), WNT-beta catenin signaling (NES = 0.78, *p* = 0.764, Figure 5C), and Hedgehog signaling (NES = 0.80, *p* = 0.718, Figure 5D) were not enriched in the LVI-positive tumors. We further investigated neuropilin-1 (NRP1) expression, as it acts as a co-receptor for TGF-beta. However, the NRP1 expression was not associated with LVI status (Figure 5E). This may be because NRP1 interacts not only with TGF-beta, but also with multiple other growth factors [38]. These findings suggest that positive LVI is associated with promoted cell cycles and enhanced TGF-beta signaling pathways, possibly leading to a worse prognosis with higher tumor growth and potential to metastasize to lymph nodes.

## 3. Discussion

To our knowledge, this is the first study evaluating the association between pathologically proven LVIs and transcriptomic information in a PDAC cohort. Some of the pathological information, such as LVI or PNI, is not included in clinicopathological data in the TCGA cohort. However, with the Text Information Extraction System (TIES), we were able to access the de-identified original pathology reports on each TCGA patient and link pathological information to the transcriptomic data manually. This methodology enabled us to complete this study.

Similar to our previous study on the association between enhanced vascularity in PDACs and better survival, we investigated the association between LVI-positive PDACs and lymphangiogenesis and ECM degradation, utilizing transcriptomic profiling analysis. We found that PNI status and AJCC T and N categories were associated with LVI status in the PDACs. Using the TCGA cohort, we confirmed that positive LVI status was significantly associated with poor survival in PDAC and an independent risk factor for OS, echoing the previous reports [14,15]. Further, transcriptomic investigation revealed that the PDACs with positive LVI were associated with enhanced cell cycles and promoted TGF-beta signaling pathways, but not with EMT or lymphangiogenesis.

We found that that positive LVI tumors were associated with a higher AJCC T category and increased lymph node metastases, which is in agreement with previous studies [5,7,14]. Epstein et al. reported that PDACs with positive LVI have higher potential to metastasize to the regional lymph nodes through the lymphatic channels, yet LVI is also a significant risk factor for survival independent of lymph node metastasis [14]. Although the tumor size between the LVI-positive and negative tumors was similar, LVI-positive tumors appeared to be more proliferative. As commonly seen in clinical practice, small PDACs do have the ability to metastasize to lymph nodes as well as distant organs, which makes it conceivable that tumor size is only one aspect of tumor aggressiveness. LVI is a complicated invasion process, requiring tumor cells to detach themselves from the growing primary mass, followed by invasion and migration through the ECM towards vascular walls. In PDACs, VEGFs were reported to associate with enhanced angiogenesis and lymphangiogenesis [39,40]. Additionally, in breast cancers, a strong correlation has been reported between LVI and genes controlling ECM degradation, angiogenesis, neovascularization, and tumor necrosis factor [28,29,31]. Due to scarce evidence of transcriptomic characterization with LVI in PDACs, we investigated the association between LVI status and these previously reported genes, which unexpectedly revealed no association in PDACs. Furthermore, EMT is known to be one of the major mechanisms of PDAC metastasis, as the transformation is associated with increased mobility of the cancer cells [41]. Transcriptomic profiles in the present study demonstrated no association between EMT and LVI status; however, this may be because EMT activation might have occurred in an early phase during pancreatic cancer initiation [42], whereas LVI-positive tumors are in advanced PDACs. Positive LVI was associated with enhanced cell cycles, such as mitotic spindle and G2/M checkpoint gene upregulation. These results suggest that cancer cells with enhanced cell cycles and rapid proliferation [43] lead to lymphovascular invasion and subsequent lymph node metastases. Interestingly, our group recently found that breast cancer with LVI similarly demonstrated enhanced cellular proliferation independent of lymphangiogenesis, which suggests that LVI might be simply a marker of tumor proliferation with worse tumor biology, rather than increased lymphangiogenesis [44]. We cannot help but speculate that EMT and lymphangiogenesis may not be as clinically relevant as tumor proliferation based on these results.

TGF-beta signaling was associated with positive LVI status in the present study. TGF-beta signaling is one of several core signaling pathways commonly altered in PDACs [36], and enhanced expression is associated with poor prognoses [45,46]. TGF-beta mediates interaction between pancreatic stellate cells and cancer cells, producing a rich stromal reaction, which is one of the major characteristics of PDACs [47]. TGF-beta signaling also regulates immune cells in TME, inhibits the functions of natural killer (NK) and CD8+ T cells, induces pro-oncogenic regulatory T cells (Treg), and inhibits B cell proliferation [48]. Additionally, Bierie highlighted a paracrine mechanism of TGF-beta, interacting between various cell populations in TME and playing a significant role in promoting cell proliferation by secreting growth factors and increasing metastatic potential [49]. Interestingly, TGF-beta is also known to have a dual role in PDAC progression: a pro-oncogenic role and an anti-oncogenic role [45]. While TGF-beta acts as a tumor suppressor in early-stage pancreatic cancer by inhibiting epithelial cell cycle progression and promoting apoptosis, it becomes pro-oncogenic in later stages by promoting EMT, inducing pro-oncogenic immune cells in TME, increasing cell motility, and increasing distant metastasis [45,50].

These novel findings should be noted in the context of the limitations of this study. First, we utilized solely publicly available TCGA datasets. We are aware that our findings and conclusions would have been stronger with a validation cohort. Unfortunately, given the low incidence of pancreatic cancer despite its high mortality, a cohort including transcriptomic and clinicopathological data was not available, despite our extensive search. Second, there was some missing information from the original reports in TIES. However, we were able to retrieve approximately 85% of LVI and PNI statuses. Further, pathological information for LVI and PNI was obtained from the original reports through TIES, and these reports were not validated or standardized [51]. Third, whereas TCGA has significant benefits, with a large amount of transcriptomic information associated with patient survival, it also has limitations, such as a relatively short follow-up duration, as well as no detailed information available for neoadjuvant or adjuvant chemotherapy. Additionally, the cohort only contains surgically resected specimens, not metastatic sites, which could possibly make our conclusion difficult to generalize. In addition, we did not discuss developing definitive biomarkers to differentiate patients with a high risk of LVI, since the aim of this study was to elucidate the underlying cancer biology of LVI. Development of a new biomarker to differentiate PDAC patients with worse outcomes will be interesting and will be pursued in a different manuscript. Lastly, this study does not include any in vitro or in vivo experiments; therefore, all of our findings were observational associations. To this end, our study did not prove any causal mechanisms; however, since this study elucidated the underlying biology of LVIs using transcriptomic profiles from actual human pancreatic cancer, this study is more precise than in vivo or in vitro experiments in terms of reflecting human cancer. Despite these limitations, we believe that this study still provided valuable underlying aggressive biological information on LVI-positive PDACs.

## 4. Materials and Methods

### 4.1. Data Acquisition from TCGA

Clinicopathological data and gene expression data from RNA sequences were obtained from the TCGA pancreatic cancer cohort (PAAD) through cBioPortal [52,53], which contained 185 primary pancreas tumors, as described in the past [54,55,56]. One hundred fifty-four patients were registered as “Pancreas-Adenocarcinoma Ductal Type” within OS data [57,58]. The median follow-up period was 15 months (Inter-quartile range (IQR): 8–21 months). Gene expression data were utilized after being log2 transformed. LVI and PNI information associated with the TCGA cohort was manually obtained via the TIES system that attaches the pathological reports to the TCGA cohort (http://ties.dbmi.pitt.edu/#) through the Roswell Park Comprehensive Cancer Center. Given that TCGA is a publicly available, de-identified database, the Institutional Review Board (IRB) was exempted.

### 4.2. GSEA

GSEA was performed, comparing tumors that were LVI-positive and negative, utilizing the Hallmark gene sets [59] with the software provided by the Broad Institute (https://software.broadinstitute.org/gsea/index.jsp), as described before [21,22,23,24,25,26,27,60,61].

### 4.3. Survival Analysis

DFS was defined as the time from the date of surgery to the date of disease progression. OS was defined as the time from the date of surgery to the date of death by any cause. In order to obtain hazard ratios (HRs) and 95% confidence intervals (CIs), univariate and multivariate analyses for OS were conducted, using age, gender, tumor size, AJCC Staging T and N categories, histological grade, residual tumor status, history of adjuvant radiation treatment, PNI, and LVI. AJCC pathological stage IA and IB and stage IIA and IIB were simplified to stage I and stage II, respectively. Some of the parameters were dichotomized as follows: age below 65 and age 65 and above, tumor size below 3.5 cm and greater than or equal to 3.5 cm (the median value), AJCC T category T1 + T2 and T3 + T4, pathological AJCC stage I + II and III + IV, residual tumor status R1 + R2 and R0, and histologic grade G3 (poorly differentiated) and G1 + G2 (well-differentiated + moderately-differentiated). Multivariate analysis was performed with variables with a *p*-value < 0.05 on univariate analyses.

### 4.4. Statistical Analysis

Statistical comparisons of the clinicopathological parameters were performed by a Chi square test. Continuous values were compared by a Student’s t-test. All statistical analyses were performed using JMP 14.2 (SAS, Cary, NC, USA) and R software (http://www.r-project.org/) together with Bioconductor (http://bioconductor.org/). A *p*-value < 0.05 (two-tailed) was considered to be statistically significant.

## 5. Conclusions

In conclusion, a positive LVI status was associated with shorter OS in PDACs, possibly due to accelerated cell proliferation and enhanced TGF-beta signaling, leading to faster tumor growth and higher lymph node metastasis potential. Transcriptomic profiling from human pancreatic cancer specimens elucidates more precise tumor biology in LVI-positive PDACs.

## Figures and Tables

**Table 1:** Patient Demographics.

Variables	LVI (+) (*n* = 85)	LVI (−) (*n* = 45)	*p* Value
Age (Median (IQR))	67 (60–75)	65 (57–73)	0.201
Sex (M/F)	47/38	29/16	0.312
Tumor location (Head/Body and Tail)	72/12	39/5	0.640
Residual tumor (R0/R1/R2)	43/29/3	32/10/0	0.050
Radiation treatment adjuvant (Yes/No)	14/53	6/24	0.920

IQR = inter-quartile range.

**Table 2:** Univariate/multivariate analyses (COX proportional hazards model) for OS.

	Univariate Analysis			Multivariate Analysis		
Variables	*p* Value	Hazard Ratio	95% CI	*p* Value	Hazard Ratio	95% CI
Age (≥ 65)	0.750	1.053	0.763–1.451			
Sex (M)	0.875	0.975	0.709–1.344			
Tumor size (≥ 3.5 cm)	0.530	0.898	0.641–1.256			
AJCC T (T3 + T4)	0.717	0.983	0.430–1.635			
AJCC N (N1)	0.08	1.556	0.950–2.691			
Histologic grade (G3)	0.124	1.412	0.909–2.192			
Residual tumor status (R1 + R2)	0.021	1.703	1.084–2.677	0.125	1.580	0.881–2.834
Adjuvant radiation (Yes)	0.002	0.403	0.200–0.736	0.017	0.325	0.129–0.817
Perineural invasion (PNI +)	0.282	1.472	0.747–3.342			
Lymphovascular invasion (LVI +)	0.011	1.883	1.150–3.216	0.012	2.401	1.218–4.759

## Supplementary Materials

The following are available online at https://www.mdpi.com/2072-6694/12/8/2033/s1, Figure S1: Gene expression comparison involved in cancers stem cell regulation and maintenance genes between the LVI-negative and -positive tumors.
