# Supplementary material for: Transcriptomic Profile of Lymphovascular Invasion, a Known Risk Factor of Pancreatic Ductal Adenocarcinoma Metastasis
Source: Cancers (Basel). 2020 Jul 24;12(8):2033. doi: 10.3390/cancers12082033 (PMC7465682; doi:10.3390/cancers12082033)
Supplement: Supplementary file 1 [file cancers-12-02033-s001.pdf]

Supplementary Materials

Transcriptomic Profile of Lymphovascular Invasion, a Known Risk Factor of Pancreatic Ductal AdenocarCinoma Metastasis

Hideo Takahashi, Eriko Katsuta, Li Yan, Yoshihisa Tokumaru, Matthew H.G. Katz, and Kazuaki Takabe

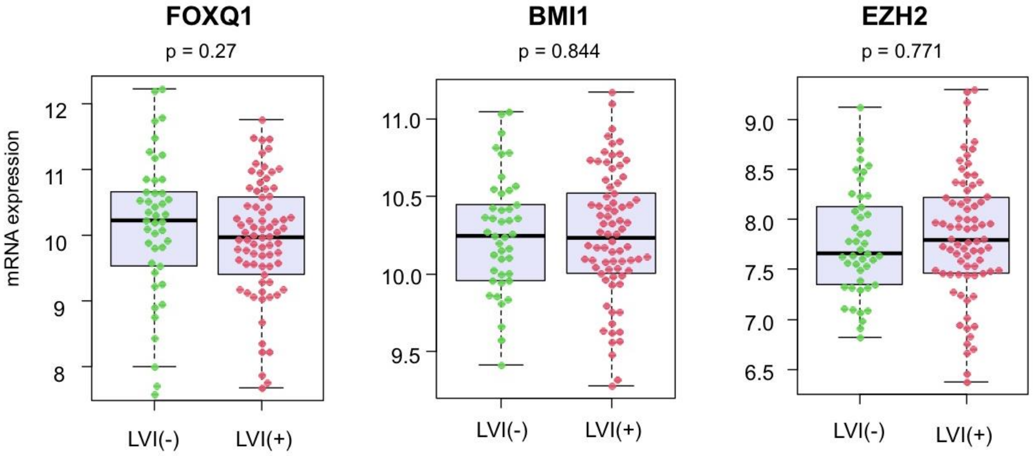

Figure S1. Gene expression comparison involved in cancer stem cell regulation and maintenance genes between the LVI-negative and -positive tumors. *LVI*, lymphovascular invasion; *FOXQ1*, Forkhead box protein Q1; *BMI1*, B cell-specific Moloney murine leukemia virus integration site 1; *EZH2*, Enhancer of zeste homolog 2.

© 2020 by the authors. Licensee MDPI, Basel, Switzerland. This article is an open access article distributed under the terms and conditions of the Creative Commons Attribution (CC BY) license (http://creativecommons.org/licenses/by/4.0/).
